# The anti-neoplastic effects of metformin modulate the acquired phenotype of fibroblast cells in the breast cancer-normal fibroblast co-culture system

**DOI:** 10.32604/or.2023.043926

**Published:** 2024-02-06

**Authors:** SAMANEH MOSTAFAVI, ZUHAIR MOHAMMAD HASSAN

**Affiliations:** Department of Immunology, Faculty of Medical Sciences, Tarbiat Modares University, Tehran, Iran

**Keywords:** Caveolin 1, Lactic acid, Metformin, NIH 3T3 cells, Neoplasms

## Abstract

Intracellular communications between breast cancer and fibroblast cells were reported to be involved in cancer proliferation, growth, and therapy resistance. The hallmarks of cancer-fibroblast interactions, consisting of caveolin 1 (Cav1) and mono-carboxylate transporter 4 (MCT4) (metabolic coupling markers), along with IL-6, TGFβ, and lactate secretion, are considered robust biomarkers predicting recurrence and metastasis. In order to promote a novel phenotype in normal fibroblasts, we predicted that breast cancer cells could be able to cause loss of Cav1 and increase of MCT4, as well as elevate IL-6 and TGFβ in nearby normal fibroblasts. We created a co-culture model using breast cancer (4T1) and normal fibroblast (NIH3T3) cell lines cultured under specific experimental conditions in order to directly test our theory. Moreover, we show that long-term co-culture of breast cancer cells and normal fibroblasts promotes loss of Cav1 and gain of MCT4 in adjacent fibroblasts and increase lactate secretion. These results were validated using the monoculture of each group separately as a control. In this system, we show that metformin inhibits IL-6 and TGFβ secretion and re-expresses Cav1 in both cells. However, MCT4 and lactate stayed high after treatment with metformin. In conclusion, our work shows that co-culture with breast cancer cells may cause significant alterations in the phenotype and secretion of normal fibroblasts. Metformin, however, may change this state and affect fibroblasts’ acquired phenotypes. Moreover, mitochondrial inhibition by metformin after 8 days of treatment, significantly hinders tumor growth in mouse model of breast cancer.

## Introduction

The interactions of tumors with stromal cells (especially fibroblasts) are associated with the acquisition of many malignant characteristics, such as drug resistance and metastasis. Fibroblasts are the most important stromal cells that were found in most of the failed therapies [1]. For instance, it has been shown that fibroblasts dramatically enhance the growth rate of tumor cells and that indirect co-culture of fibroblasts with human oral squamous cell carcinoma prevented metformin-derived apoptosis [2]. The contribution of fibroblasts and breast cancer in producing metabolic compartments and cross-feeding (the reverse Warburg effect) was reported to be involved in cancer proliferation, growth, and therapy resistance [3]. It has been proven that the main metabolic coupling phenotype of cancer-associated fibroblasts (CAF) is a lack of Cav1 and a gain of MCT4 [4,5]. On the other hand, the cytokine secretion pattern of adjacent cells may change [6]. Since fibroblasts are the primary producers of IL-6, an increase in IL-6 levels is one of the key indicators of a change in fibroblast activity, which has a significant impact on tumor growth and therapeutic resistance. Inhibiting IL-6 production enhanced treatment responsiveness in patients with high circulating IL-6 levels who had a poor prognosis [7,8].

Moreover, the studies demonstrated that TGFβ in TME contributes to the loss of Cav1 and increase in MCT4 in normal fibroblasts, leading to the acquisition of metabolic coupling and cross-feeding phenotypes [9–11]. Furthermore, it was shown that TGFβ has impaired mitochondrial function and oxidative phosphorylation (OXPHOS), thereby enhancing glycolysis and reactive oxygen species (ROS) generation. An increase in ROS and metabolic reprogramming toward glycolysis, in turn, increase lactate and induce an acidic tumor microenvironment [12]. Lactate is transported into the tumor cytoplasm by activated fibroblasts, where it promotes tumor growth and proliferation [13]. Lactate is transported into the tumor cytoplasm by activated fibroblasts, where it promotes tumor growth and proliferation [13]. It was indicated that MCT4 expression is increased in stromal fibroblasts [3]. Cross-feeding of tumor fibroblasts is known as metabolic symbiosis and the reverse Warburg effect [14].

Cav1 is a 22-kDa protein encoded by the Cav1 gene known to regulate cholesterol distribution, signal transduction, cell migration, metabolism regulation, and endocytic vesicular trafficking [9]. Poor prognosis is associated with the loss of stromal Cav1 in the fibroblast of ductal carcinoma *in situ* (DCIS), gastric cancer, and prostate cancer [10,15,16]. Therefore, based on the literature, loss of Cav1 and gain of MCT4, along with a significant increase in IL-6, TGFβ, and lactate secretion, are considered primary factors in the acquisition of a new phenotype in adjacent fibroblasts [17,18].

On the other hand, metformin, a widely used drug for type 2 diabetes, has exhibited promising anticancer properties in recent years [19]. Metformin and oxidative phosphorylation (OXPHOS) restriction of mitochondrial respiratory chain complex I promotes a metabolic stress signal in cancer cells, activating the LKB1-AMPK pathway, which inhibits protein synthesis and results in a global decrease in ATP-consuming processes, which inhibits tumor proliferation [20]. A growing body of research has highlighted the anti-cancer effects of metformin [21]. However, as a bioenergetic drug, its effects on metabolic coupling markers (Cav1, MCT4) and the factors involved in tumor-fibroblast interactions (IL-6, TGFβ, and acid lactic) are not thoroughly and simultaneously examined. Furthermore, the co-culture of NIH3T3 (mouse-derived normal fibroblast) with 4T1 cells, which are obtained from the mammary gland tissue of BALB/c mice and are capable of mimicking stage IV of human breast cancer, has not yet been examined. Here, we sought to determine: 1) whether breast cancer cells can induce a loss of Cav1 and a gain of MCT4 in normal adjacent fibroblasts; 2) whether this system affects the secretion of TGFβ, IL-6, and lactate; 3) whether this new phenotype is modulated by metformin; and 4) whether mitochondrial inhibition (OXPHOS) by metformin can reduce tumor proliferation in a mouse model of breast cancer. Also, whether the effective dose of the drug in the mouse model is well tolerated by the mice without harming the function of other organs. To this end, we performed co-culture studies using a murine breast cancer cell line, 4T1, and a normal fibroblast cell line, NIH3T3, as well as an *in vivo* evaluation of the effect of metformin on cancer growth.

## Materials and Methods

### Reagents and antibodies

Roswell Park Memorial Institute (RPMI) 1640, Dulbecco’s Modified Eagle Medium (DMEM) medium, Heat-inactivated fetal bovine serum, pen-strep, and TRIzol were purchased from, Gibco (Thermo Fisher Scientific, Waltham, Massachusetts, USA). 4′,6-diamidine-2′-phenylindole dihydrochloride (DAPI), 1,1-Dimethylbiguanide hydrochloride, Metformin, dimethyl sulfoxide (DMSO), and 3-(4,5-Dimethylthiazol-2-yl)-2, 5-diphenyl tetrazolium bromide (MTT) were obtained from Sigma-Aldrich (Burlington, Massachusetts, USA). Anti-caveolin-1 (#PA1064) was purchased from Thermo Fisher Scientific (Waltham, Massachusetts, USA) and Anti-MCT4 (#HPA 21451) and FITC-conjugated secondary antibody was Santa Cruz Biotechnology, Dallas, Texas, USA. Antibody for Annexin V-FITC (Cat # 556547, BD Biosciences, CA, USA) and Propidium Iodide (Cat #P1304MP) was also obtained from Thermo Fisher Scientific (Waltham, Massachusetts, USA).

### Cell lines and culture conditions

The 4T1 breast cancer cell line (Cat # C604) was purchased from Pasture Institute of Iran (Tehran, Iran) and embryonic mouse fibroblast cell line NIH3T3 (Cat # IBRCC10100) was purchased from the Iranian Biological Resource Center, (IBRC, Tehran, Iran). 4T1 cells were cultured in RPMI 1640, and fibroblasts were cultured in DMEM, both supplemented with 10% heat-inactivated fetal bovine serum, penicillin (100 U/ml) and streptomycin (100 μg/ml). All cultures were maintained at 37°C in a 5% CO_2_ humidified incubator. For a long-term cell-to-cell interaction the co-cultured cells were incubated 5 days for each experiment.

### Co-culture and mono-cultures of breast cancer cells and fibroblasts

In this work, co-culture and mono-culture cell types were employed. Both kinds of cultures received an addition of 2.5 mM of metformin, and a control culture with no metformin therapy was assessed. Co-culture: NIH3T3 normal fibroblasts and 4T1 cells were co-plated in 4 well plates in 0.5 ml of complete media. 4T1 cells were plated within 2 h of fibroblast plating. During seeding, the total number of cells per well was 3 × 10^3^ (2:1 4T1 to fibroblast ratio). After 24 h of plating, the media was changed to DMEM containing 10% FBS and Pen-Strep. After 5 days of co-culture, 2.5 mM metformin was added to cell culture medium, and after 24 h of treatment, the cells were fixed and prepared for an immunocytochemistry test. Homotypic cultures of fibroblasts and 4T1 cells were plated in parallel without treatment as a control.

Mono-culture: Using the same cell number allotted to each co-culture, homotypic cultures of fibroblasts and 4T1 cells were planted simultaneously. Metformin was introduced to these cell cultures in accordance with what has already been said, and the identical cell cultures without metformin were employed as a control. Mono-cultures and co-cultures were maintained at 37°C in a 5% CO_2_ humidified incubator.

### Mouse model of breast cancer

This interventional study has been conducted in the Medical Immunology Department of Tarbiat Modares University (TMU), and the institutional ethical committee of TMU approved this protocol (No. IR.MODARES.REC.1399.093 dated 14 October 2020).

For the evaluation of the anticancer activity of metformin, we utilized 12 BALB/c female mice with ages ranging between 6 and 8 weeks (6 mice in each group). The sample size was calculated by the ‘resource equation’ technique as defined by [22]. According to [23], Group A was assigned to receive metformin 150 mg/kg/day (IP injected), and Group B was assigned to receive 30 mL/kg/day PBS (IP injected) for 14 days. The number of 5 × 10^6^ 4T1 breast cancer cells was suspended in 100 μl of PBS mixed with 50% Matrigel (BD Biosciences) and infused into the back of the right flank of the mice. Tumor development was observed using a Vernier caliper, and treatments were started when tumors reached an average size of 200 mm^3^. The anticancer activity of Metformin has been evaluated by comparing the tumor volume, tumor growth, and survival rate. The following formula was used for the calculation of the tumor volume:

V=0.5236×W×L×T

where L represents the length and W represents the Width.

**Tumor control ratio:** The formula used to calculate the tumor control ratio is as follows [24]:

$$T/C=\frac{Cancer\;Growth\;Volume\;of\;the\;Experiment\;Group}{Cancer\;Growth\;Volume\;of\;the\;Control\;group}$$

Agents generating a T/C of around 15% are considered highly active against the tumor; agents with a mean tumor volume T/C of about 45% but >15% are considered to have intermediate activity; and those with mean T/C values >45% are considered to have low activity levels.

**Tumor growth ratio (V/V0):** this ratio shows the tumor growth rate (TGR) per day of the experiment. V/V0 can be calculated by dividing the volume of the treatment group at a particular time by the preliminary tumor boom at baseline (earlier than starting the therapy) [23].

### Immunocytochemistry (ICC)

The ICC protocol was performed as previously described [10]. In brief, cells were fixed with 2% paraformaldehyde (PFA) in PBS, pH 7.4, for 20 min. Afterwards, the cells were permeabilized with 0.2% Triton X-100 for 20 min, blocked with 1% BSA solution for 45 min, and incubated with primary antibody (1:1000 µg/ml) overnight at the incubator. Upon 3 h of incubation at room temperature with the secondary antibody (1:1000 µg/ml), cells were stained with DAPI (1:1000µg/ml) for 5 min at room temperature. Cells were washed three times in PBS with a pH of 7.4 in between each step. Cells may then be examined using a fluorescent microscope (Olympus, Japan, Inverted Microscope Model IX70). Importantly, identical exposure settings were used to get the pictures. Using Image J software (version 1.50i, National Institute of Health, Bethesda, MD, USA), pictures were also analyzed.

### Evaluation of cell viability/proliferation by MTT assay

The MTT assay was performed as previously described [25]. In brief, in separate 96-well plates, 4T1 cells (5 × 10^4^ cells/well) and normal fibroblasts (1 × 10^6^ cells/well) were seeded and cultured in 200 μl RPMI. The day after, cells were treated with different concentrations of metformin for 24 h. Then, 20 μl of MTT solution containing 5 mg/ml of MTT powder was added to each well and incubated for 3 h in a 5% CO_2_ atmosphere in a 37°C incubator. Then, the supernatant was removed from all the wells, and 100 μl of DMSO was added. Afterward, the absorption was measured at 540 nm by a microplate reader. All of the samples were tested in triplicate, and the survival rate (%) was calculated using the following equation:

$$Survival\;rate\;\left(\%\right)\;=\frac{OD\;treatment\;group}{OD\;control\;group}\times 100$$

### Measurement of apoptosis with PerCP-Cy™5.5 Annexin V

PerCP-Cy™5.5 Annexin V was used to evaluate the apoptosis ratio after treatment with metformin. In brief, 4T1 cells were seeded in six-well plates at a density of 1 × 10^6^ per well in a 24-well plate cultured overnight at 37°C in a 5% CO_2_ humidified incubator. Then, the cells were treated with metformin for 24 and 48 h. The cells were then yielded, washed with PBS, suspended with a 500 μl binding buffer, added 5 μl annexin-V-FITC plus 5 μl PI, and incubated for 15 min at room temperature in a dark place. The graphs were obtained from flow cytometry (BD Biosciences), and the data was analyzed by Flowjo version 7.6.1.

### Gene expression assay

Briefly, 4T1 cells were cultured in a six-well plate at a concentration of 1 × 10^6^ cells/well for 24 h. After treating with metformin in different concentrations for 24 h, cells were harvested, and total RNA was extracted using TRIzol treatment based on the manufacturer’s protocol. Afterward, complementary DNAs were synthesized using the cDNA Synthesis Kit (Thermo Fisher Scientific) based on the manufacturer’s protocol. The expression levels of genes were determined with the StepOnePlus real-time PCR system (Applied Biosystems). An endogenous control gene (hypoxanthine guanine phosphoribosyl transferase) and the particular target gene of interest were amplified using real-time polymerase chain reactions using the set of primers in all assays. After amplification, fold-change expression analysis was carried out. Table 1 indicates the sequences of primers.

**Table 1:** Set of primers sequence

Gene	Primer sequence
P53	**Forward** GTT CCG AGA GCT GAA TGA GG
	**Reverse** ACT TCA GGT GGC TGG AGT GA
BAX	**Forward** ATG GAC GGG TCC GGG GAG
	**Reverse** ATC CAG CCC AAC AGC CGC
HPRT	**Forward** CAG GAC TGA AAG ACT TGC TC
	**Reverse** AGG TCA GCA AAG AAC TTA TAG

### IL-6 and TGFβ measurement using enzyme-linked immunosorbent assays (ELISA)

4T1 and fibroblast cells were co-cultured in 12-well plates, which were treated with metformin. Monoculture of each group of cells was used as a control. IL-6 and TGFβ concentrations in the cultured media were evaluated by the mouse IL-6 ELISA kit (ab 46100, Abcam, Cambridge, UK) and the mouse TGF beta ELISA kit (ab119557, Abcam, Cambridge, UK) according to the manufacturer’s protocol.

### Measurement of lactate production

The change in lactate level was determined by using a commercially available assay kit (Spinreact, Spain). In a nutshell, metformin was administered while 4T1 and fibroblast cells were co-cultured on a 12-well plate. Each cell group’s monoculture served as the control. Following that, lactate concentration was determined using a commercial kit in accordance with the manufacturer’s instructions at a wavelength of 505 nm (490–550). The principle of the method is based on the reactions bellow:

$$L-Lactate+O_{2}+H_{2}O\to Pyruvate+H_{2}O_{2}$$

$$2H_{2}O_{2}+4‒AP+4‒Chlorophenol\to PODQuinone+H_{2}O$$

The intensity of the colour formed is proportional to the lactate concentration in the sample.

$$\begin{array}{r} mg/dL\;lactate\;in\;the\;sample=\;\frac{\;\left(A\right)Standard\;\left(A\right)Blank\;}{\left(A\right)Sample\;\left(A\right)Blank\;}\\ \;\times 10\;\left(Standard\;conc\right) \end{array}$$

Conversion factor: mg/dL × 0,1123 = mmol/L.

*(A) refers to absorbance.

### Statistical analysis

Statistical significance was evaluated using the chi-square test, one-way *ANOVA*, and two-way *ANOVA* with GraphPad Prism 9.0 (Graph Pad Software, Inc., San Diego, CA, USA). The values of *p* < 0.05, *p* < 0.01, and *p* < 0.001 were considered statistically significant and highly significant differences, respectively.

## Results

### Metabolic coupling markers, MCT4 and Cav1, were significantly altered in co-cultured fibroblasts

**Figure 1:**
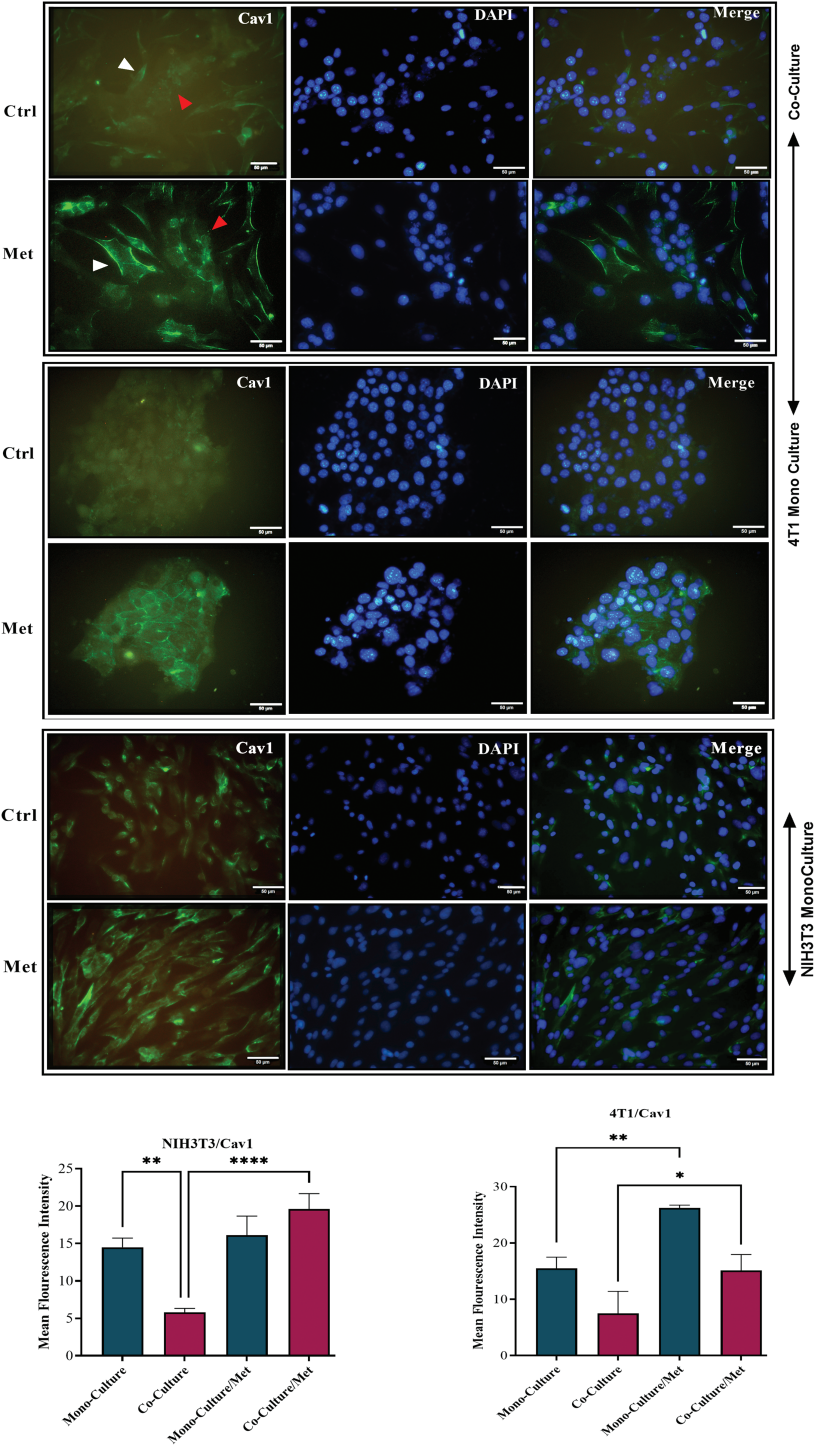
Cav1 in co-cultures and mono-cultures of 4T1 and NIH3T3 normal fibroblasts treated with metformin (Met) compared with control (Ctrl). The white arrows point at the fibroblasts, while the red arrows point at the tumor cells. Normal NIH3T3 fibroblasts and 4T1 cells were co-cultured for 5 days. Then, the cells were fixed and immunostained with antibodies directed against Cav1 (green). Nuclei were counter-stained with DAPI (blue). As controls, mono-cultures of normal NIH3T3 fibroblasts and 4T1 breast cancer cells were fixed and stained in parallel. Note that Cav1 is greatly down-regulated in fibroblasts co-cultured with 4T1 cells compared with mono-cultured fibroblasts. Cav1 was enhanced in mono-cultured 4T1 cells after treatment, while in normal fibroblasts it did not undergo a significant change. Importantly, images were acquired using identical exposure settings. Treatment with metformin, however, reversed this condition, and Cav1 was significantly re-expressed in treated co-cultured cells. *p < 0.05; ** *p* < 0.01; ****p < 0.0001.

**Figure 2:**
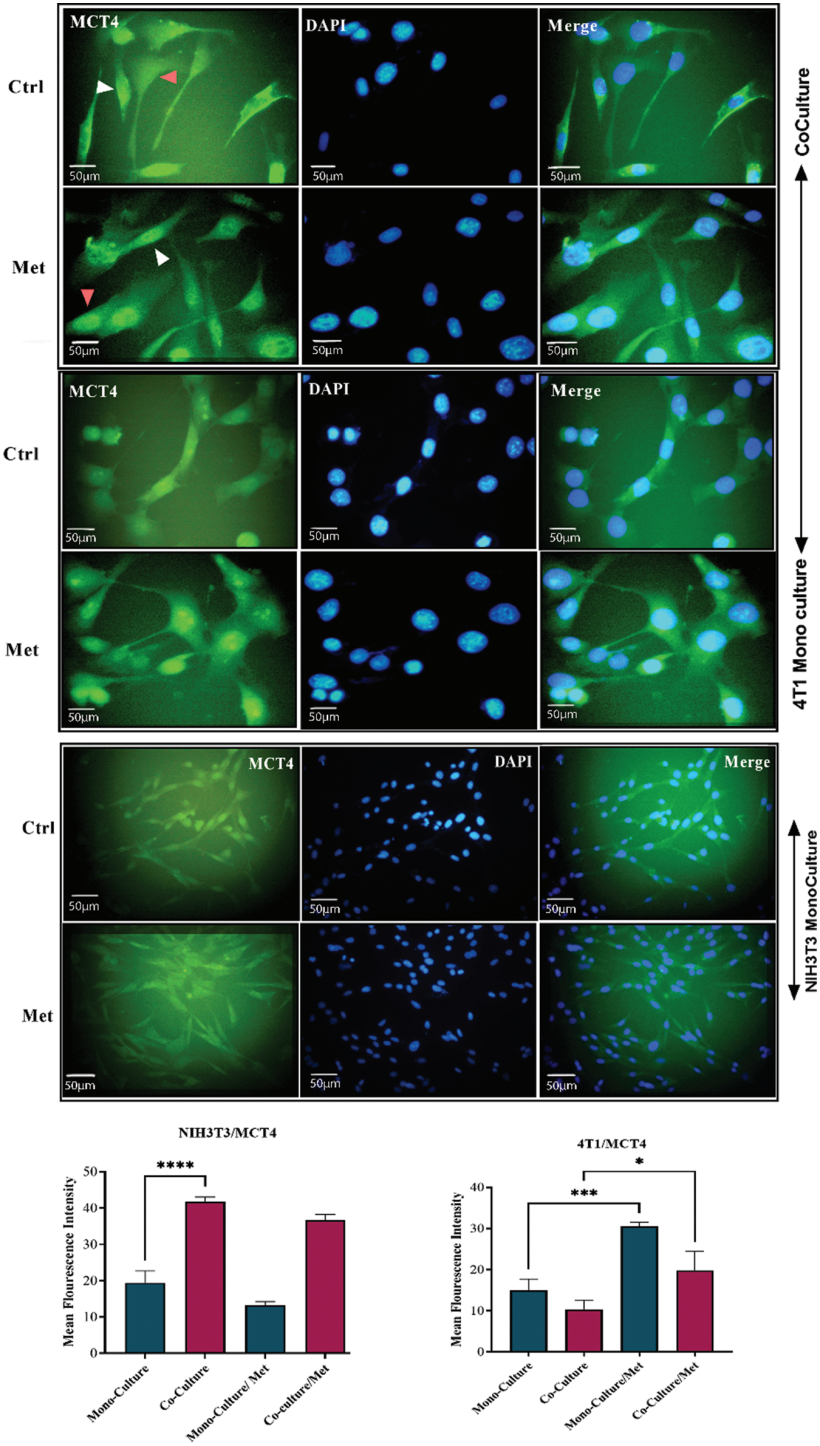
MCT4 in co-culture and monoculture of 4T1 and normal NIH3T3 fibroblasts treated with metformin (Met) compared with control (Ctrl). MCT4 is significantly increased in co-cultured NIH3T3 fibroblasts (white arrows) compared with fibroblasts in mono-cultures. Also, treatment with metformin increases MCT4 in 4T1 cells (red arrows). While MCT4 in treated 4T1 monoculture is increasing, monocultured NIH3T3 cells show a down-regulation of MCT4 in response to metformin. NIH3T3 fibroblasts and 4T1 cells were co-cultured for 5 days. Then, the cells were fixed and immunostained with antibodies directed against MCT4 (green), and nuclei were counter-stained with DAPI (blue). As controls, 4T1 and fibroblast mono-cultures were fixed and stained in parallel. Co-culture induces increase in cytokine secretion from both fibroblast and tumor. *p < 0.05; *** *p* < 0.001; **** *p* < 0.0001.

After prolonged (5 days) co-plating of NIH3T3 with 4T1, an ICC test was conducted, which demonstrated that Cav1 was decreased in NIH3T3 fibroblast cells compared with monoculture (*p* < 0.001). Cav1 of 4T1 cells decreased during co-culture (Fig. 1). Moreover, a comparison of the ICC test of MCT4 in co-culture and monoculture of fibroblasts showed that after 5 days of cell-to-cell interactions, MCT4 was significantly up-regulated in adjacent NIH3T3 cells of co-culture (*p* < 0.0001); however, it was not significant in co-cultured 4T1 cells compared with mono-culture (Fig. 2).

### Inhibition of mitochondrial complex I by metformin influenced metabolic coupling markers; Cav1 and MCT4

As shown in Fig. 1, showing the results of the ICC test of Cav1, metformin significantly re-expressed down-regulated Cav1 in co-cultured fibroblasts (*p* < 0.0001) and 4T1 cells (*p* < 0.05). Although mitochondrial dysfunction caused by metformin significantly enhanced Cav1 in mono-cultured 4T1 cells (*p* < 0.05), its effects on normal fibroblasts were not significant. As can be seen in Fig. 2, the results of the ICC test on metformin-treated wells showed that the administration of metformin caused a decrease in MCT4 of fibroblast cells in monoculture (*p* < 0.05), while in monocultured tumor cells, MCT4 was significantly increased (*p* < 0.001). Even though co-cultured fibroblasts also showed a decrease in MCT4, this decrease was not as significant as it was in normal fibroblasts. Furthermore, treated co-cultured fibroblasts seemed to be resistant to metformin and still expressed more MCT4 than treated normal fibroblasts (*p* < 0.0001). Although metformin enhanced MCT4 of cancer cells in both types of cultures and its effects were more significant on monocultured 4T1 than on co-culture, the differences between these two cultures for tumor cells were not significant.

**Figure 3:**
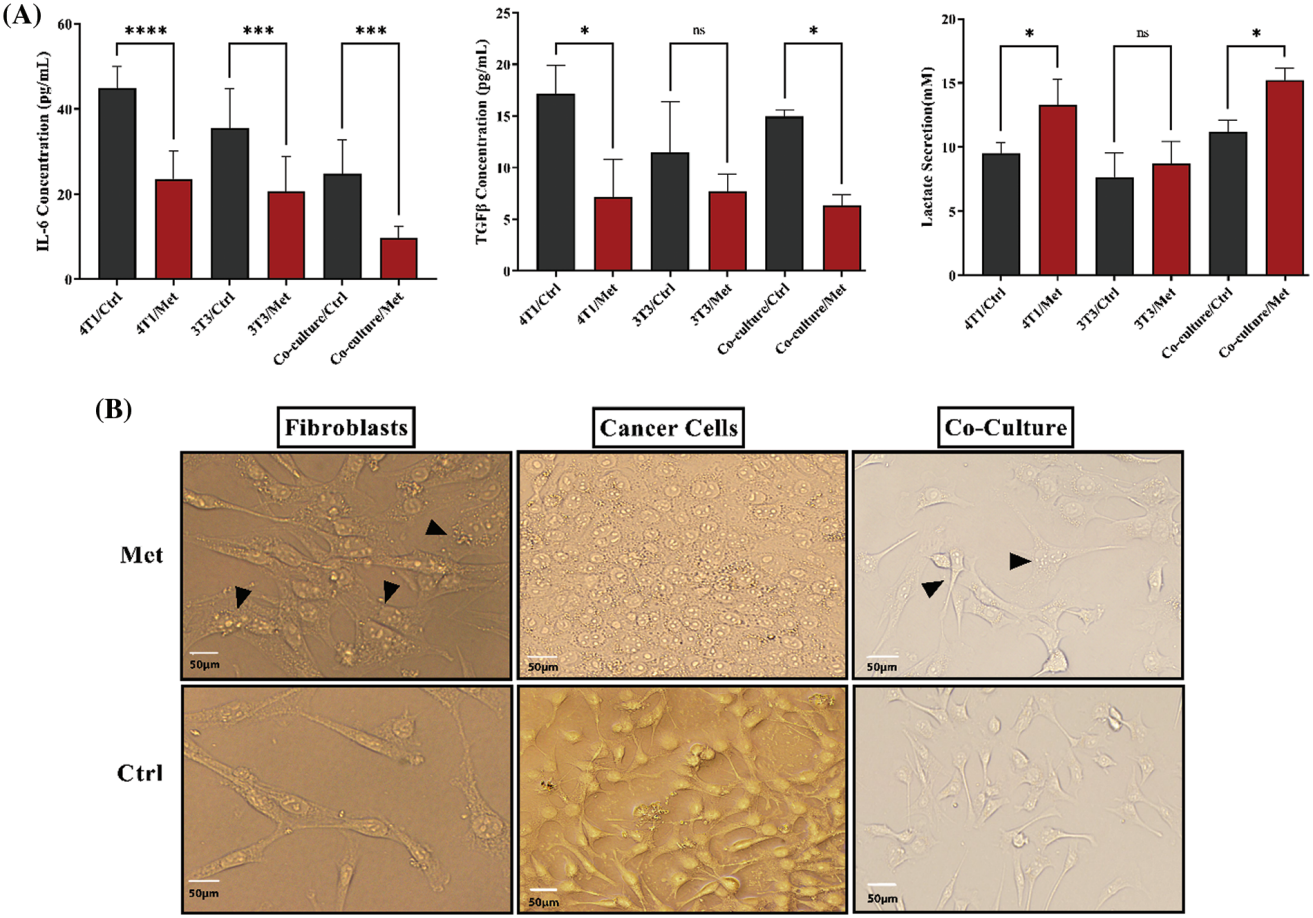
IL-6, TGFβ, and lactate in both co-culture and monoculture were modulated by metformin, and fibroblasts decreased the generation of stress granules in treated breast cancer cells. (A) After performing the cultures, cells were treated with metformin to evaluate the effects of treatment on cytokine secretion. Metformin significantly decreases IL-6 and TGFβ, although co-culture relatively increases the secretion of TGFβ. Moreover, IL-6 secretion is higher than TGFβ in mono cultures. Co-culturing slightly increases lactate secretion; however, treatment with metformin significantly increases acid-lactic levels. (B) Metformin-induced stress granules accumulate more in treated monocultures than in co-cultures. The images of both types of cultures show that the stress granules of co-cultured cells are lower than those of monocultures. ns *p* > 0.05; *p < 0.05; *** *p* < 0.001; **** *p* < 0.0001.

TGFβ and IL-6 are mainly secreted by fibroblasts; hence, we decided to investigate if the co-culture of 4T1 and NIH3T3 normal fibroblast could affect cytokine secretion level. ELISA tests showed that the levels of both cytokines and lactate were increased after prolonged co-culture. While, after treatment with metformin TGFβ and IL-6 were significantly down-regulated (*p* < 0.05), lactate levels in 4T1 mono-culture, and co-culture wells stayed high and even increased compared with untreated cells (*p* < 0.05) (Fig. 3A). Moreover, co-culture of fibroblast cells decreased stress granules formation caused by metformin in breast cancer cells (Fig. 3B).

### Metformin efficiently inhibits tumor growth and induces apoptosis after 24 h of treatment

**Figure 4:**
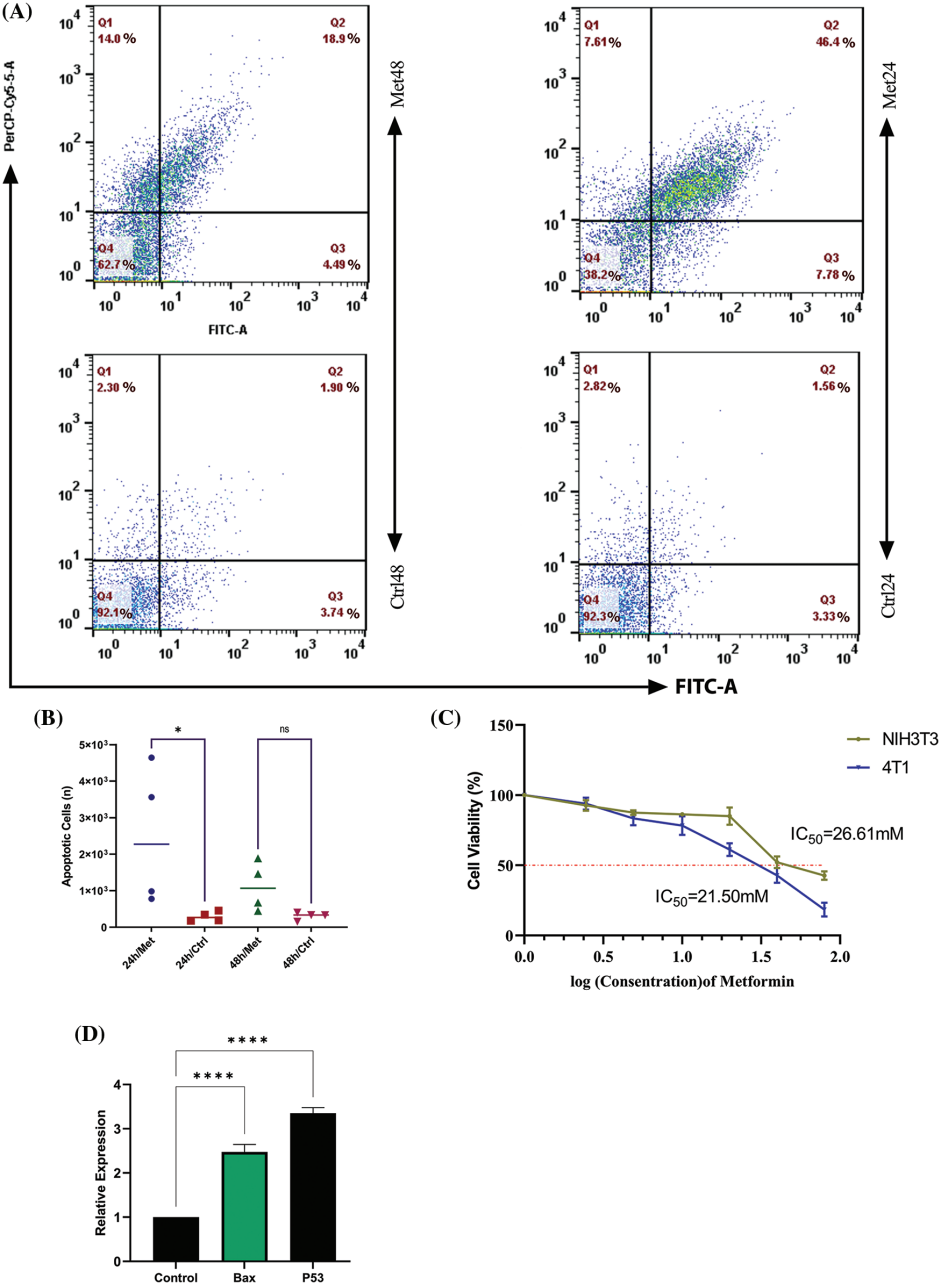
Metformin enhances the apoptosis rate in 4T1 breast cancer cells. (A) Flow cytometry of 4T1 during 24 and 48 h of treatment shows that the apoptosis rate is 46.4% in the first 24 h of treatment, while with the passage of time, the rate of apoptosis decreases to 18.9%. (B) The statistical analysis graph of flow cytometry results during 24 and 48 h of treatment. (C) The MTT assay of 4T1 shows the sensitivity of breast cancer cells to mitochondrial disruption caused by metformin compared with normal fibroblasts. (D) BAX and P53 gene expression increase after treatment with metformin, which confirms the flow cytometry results. ns *p* > 0.05; *p < 0.05; **** *p* < 0.0001.

To see whether bioenergetic dysfunction caused by metformin diminishes breast cancer proliferation, we measured cell apoptosis using PerCP-Cy™5.5 Annexin V flow cytometry test. The results showed a significant increase in apoptosis rate after 24 h of treatment with metformin (44.1% *p* < 0.05); however, by the passage of time until 48 h from the treatment apoptosis rate decreased to 18.9% (Figs. 4A and 4B).

### Breast tumor cells are more sensitive to mitochondrial dysfunction caused by metformin than fibroblasts

The MTT test findings demonstrated that 4T1 cell viability dramatically declined (*p* < 0.05) with increasing drug concentration up to a dosage of 40 mM, at which point 50% of 4T1 cells were dead. However, NIH3T3 normal fibroblast cell viability remained roughly constant at doses of 20 and 40 mM (Fig. 4C).

### Metformin significantly increases tumor suppressor and apoptosis-related gene expression in breast cancer cells

After 24 h of treatment, 4T1 cells were subjected to a Real-Time PCR assay for evaluating the P53 and BAX gene expression. The results of this experiment showed that mitochondrial inhibition through metformin significantly increased P53, known as a tumor suppressor gene, and consequently, BAX, which helps induce apoptosis. Therefore, metformin by this gene reprogramming enhances tumor cell death within 24 h after treatment (*p < 0.00001*) (Fig. 4D).

### Mitochondrial inhibition by metformin effectively inhibited tumor growth in mouse model of breast cancer after around 8 days of treatment

**Figure 5:**
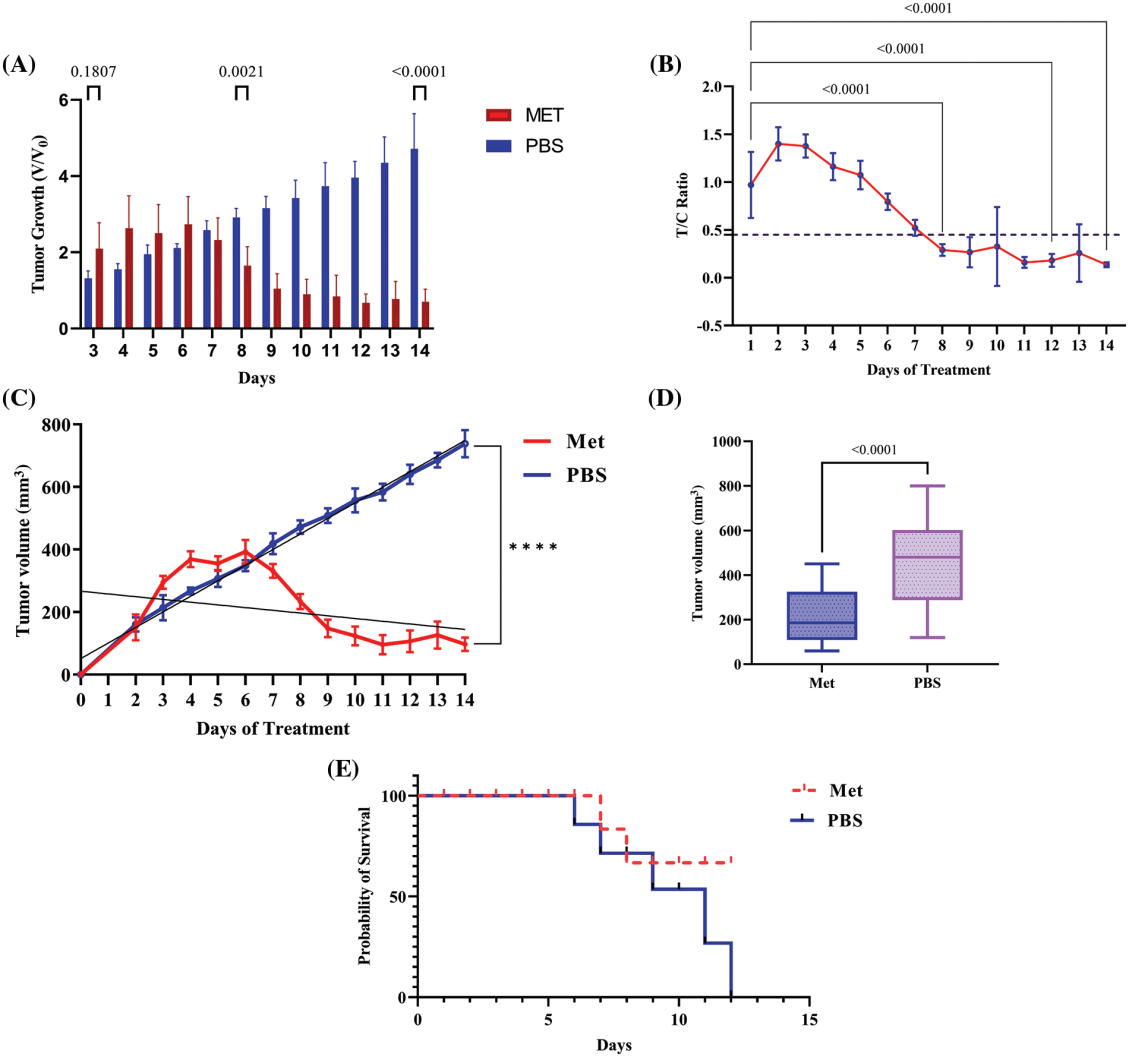
Metformin induces tumor shrinkage and rescues tumor-bearing mice. (A) V/V_0_ graph, which shows the result of dividing the volume of the treatment group at a particular time by the preliminary tumor boom at baseline demonstrated significant decrease in tumor volume in treated group. (B) After around 8 days of treatment with 150 mg/kg metformin, tumor growth began to inhibit, and the T/C ratio decreased after this time to less than 45%. (C) The regression analysis graph of tumor volume (mm^3^). (D) The graph of tumor volume containing whole the samples as a one box. (E) Mice with no treatment died after 12 days; however, the mortality rate in treated mice was lower. **** *p* < 0.0001.

The comparison of tumor volume between both groups revealed that, compared with the control group, metformin significantly reduced tumor growth (V/V_0_) in the 4T1 transplant model of mice (*p* < 0.0001). As can be seen in Fig. 5, the T/C ratio, which indicates the efficacy of the drug, decreased on day 8 of treatment to less than 45% (*p* < 0.0001). However, after 6 days of not treating the tumor, the mortality rate increased in the mice that had the tumor. Whereas, in mice receiving an IP injection of 150 mg/kg metformin, the mortality rate decreased and tumor volume shrank to less than 100 mm^3^ on the last day of the experiment (*p* < 0.00001).

## Discussion

In the present study, we used fibroblast-breast cancer co-culture to evaluate the potential effects of cancer cells on normal fibroblasts. However, the probable effects of metformin on tumor-fibroblast cross-talk were assessed. Based on the previous studies, Cav1, MCT4, acid lactic, TGFβ, and IL-6 are crucial hallmarks of cancer, involving tumor progression and therapy resistance via promoting metabolic symbiosis [4,14,26].

Cav1 and MCT4 are known metabolic coupling markers in tumor-fibroblast interactions, and fibroblasts in the tumor microenvironment undergo loss of Cav1 and gain of MCT4 [27]. It has been shown that raising the oxidative stress of TME, which in turn increases autophagic Cav1 degradation, is one of the primary causes of Cav1 depletion in stromal cells. Two pro-autophagic transcription factors, HIF-1α and NF-κb, explain the main molecular mechanisms behind this issue. CAV1 null CAFs are highly glycolytic and significantly increase lactic acid generation; hence, it is expected that MCT4 expression also increases in Cav1 null cells [3]. Moreover, a lack of Cav1 impairs mitochondrial OXPHOS and imposes glucose on aerobic glycolysis, thereby increasing lactate generation [28]. MCT4, however, is responsible for releasing monocarboxylates (e.g., lactate, ketone bodies, and pyruvate) and contributes to cross-feeding [19]. Lactate and other monocarboxylates leave fibroblasts during cross-feeding and enter the tumor cytoplasm through MCT4, where they contribute to the metabolism of the tumor and ATP synthesis. Numerous cancers have a bad prognosis when stromal MCT4 levels rise, according to studies [29,30].

Our findings from the co-culture model described here revealed that the cellular interactions between 4T1 mammary carcinoma cells and normal fibroblasts drive the loss of Cav1 and the gain of MCT4 in normal fibroblasts. Studies indicated that the increase in MCT4 is associated with a poor prognosis in breast cancer patients, and the absence of stromal Cav1 indicated early tumor recurrence and poor clinical outcomes [31,32]. On the other hand, breast cancer patients who received metformin showed a better prognosis [33]. Moreover, studies indicated that metformin by increasing Cav1 enhanced the efficacy of trastuzumab emtansine (T-DM1), an antibody drug conjugate, in HER-2-positive metastatic breast cancer [34].

Co-culture of 4T1 cells with fibroblasts also resulted in less stress granule generation after treatment (Fig. 3B). In addition, an increase in IL-6, TGFβ, and lactate secretion after prolonged co-culture indicated a small part of the fact that intracellular communications between cancer cells and fibroblasts play a key role in promoting the normal fibroblasts to acquire new secretion phenotypes. On the other hand, metformin may reverse the present condition by down-regulating IL-6 and TGFβ and re-expressing Cav1 in fibroblasts. As previously shown, metformin inhibits fibroblast differentiation in the tumor microenvironment and blocks TGFβ signaling [35,36]. TGFβ, however, by increasing IL-6 and MCT4, as well as down-regulating Cav1, is known as the most important cytokine contributing to carcinoma-associated fibroblast differentiation [36].

Furthermore, epithelial-mesenchymal transition and metastatic dissemination are both significantly influenced by fibroblast-derived IL-6 [37]. Compensatory stimulation of glycolysis (lactate) to make ATP is a result of metformin-mediated inhibition of mitochondrial complex I, which increases acid lactic (acidosis) and MCT4. As tumor cells are deeply dependent on mitochondrial-mediated ATP for their rapid growth and substitution of OXPHOS with glycolysis may produce fewer ATP, inhibiting mitochondria by metformin promotes a pivotal challenge for tumor proliferation [38]. Thus, our study demonstrated that an increase in Cav1, MCT4, and lactate (glycolysis) in metformin-treated 4T1 cells was simultaneous with an enhancement in BAX, P53, and apoptosis rate.

In this regard, an animal model of breast cancer demonstrated that mitochondrial inhibition may not be a promising drug in the first week of treatment (unlike *in vitro* models, in which metformin effectively inhibited tumor growth in just 24 h). An *in vivo* study demonstrated that after around 8 days of treatment, metabolic stress caused by metformin can hinder tumor growth. It shows that metabolic targeting may not instantly affect tumor growth, and OXPHOS is a fundamental and crucial pathway for cancer proliferation. Hence, targeting OXPHOS in cancer cells may be a promising attitude toward cancer therapy. A previous study on MCF-7 cells and a total sample size of 50 athymic BALB/c mice showed that on day 7 of treatment, the tumor size of all the samples reached 100 mm^3^ [23], just like what our results indicate.

Our *in vitro* results also indicated that although during the first 24 h and immediately after treatment, the apoptosis rate significantly increased, during the second day of treatment, the apoptosis rate was down-regulated, indicating that metformin is no longer cytotoxic for rescued tumor cells. The metabolic stress brought on by metformin seems to have been overcome by these cells. The half-life of metformin is 12 h, although it is anticipated that the medicine would become less effective beyond 48 h of therapy. Therefore, metformin-mediated mitochondrial dysfunction and, as a consequence, energetic stress (low ATP/AMP) may promote a crucial cytotoxic condition for cancer cells; however, adaptation to the current rough metabolic situation is also probable. As a result, metformin may not always be cytotoxic to cancer cells, and those cells may continue to respond to metformin over the long term by replenishing ATP and metabolite levels [39]. Our research shows that when breast cancer and normal fibroblast cells are co-cultured, substantial phenotypic and secretory alterations may occur. Moreover, metformin reversed the condition and modulated the acquired phenotypes of fibroblasts.

However, this study contains some shortage, for instance the potential mechanism by which metformin reverses the phenotype of fibroblast cells in the breast cancer-normal fibroblast co-culture system needs to be further studied in future research. Moreover, performing such studies on human samples seems to be worthwhile.

## Data Availability

All data generated or analyzed during this study is included in this published article.

## References

[1] Ni, Y., Zhou, X., Yang, J., Shi, H., Li, H. et al. (2021). The role of tumor-stroma interactions in drug resistance within tumor microenvironment. Frontiers in Cell and Developmental Biology*,* 9*,* 637675; 34095111 10.3389/fcell.2021.637675 PMC8173135

[2] Zhang, Z., Liang, X., Fan, Y., Gao, Z., Bindoff, L. A. et al. (2019). Fibroblasts rescue oral squamous cancer cell from metformin-induced apoptosis via alleviating metabolic disbalance and inhibiting AMPK pathway. Cell Cycle*,* 18 *(*9*),* 949–962; 31014173 10.1080/15384101.2019.1598727 PMC6527302

[3] Wilde, L., Roche, M., Domingo-Vidal, M., Tanson, K., Philp, N. et al. (2017). Metabolic coupling and the reverse warburg effect in cancer: Implications for novel biomarker and anticancer agent development. Seminars in Oncology*,* 44 *(*3*),* 198–203; 29248131 10.1053/j.seminoncol.2017.10.004 PMC5737780

[4] Raudenska, M., Gumulec, J., Balvan, J., Masarik, M. (2020). Caveolin-1 in oncogenic metabolic symbiosis. International Journal of Cancer*,* 147 *(*7*),* 1793–1807. 32196654 10.1002/ijc.32987

[5] Zhai, Y., Chai, L., Chen, J. (2017). The relationship between the expressions of tumor associated fibroblasts Cav-1 and MCT4 and the prognosis of papillary carcinoma of breast. Pakistan Journal of Pharmaceutical*,* 30 *(*Suppl 1*),* 263–272. 28625953

[6] Sun, C., Wang, S., Zhang, Y., Yang, F., Zeng, T. et al. (2021). Risk signature of cancer-associated fibroblast-secreted cytokines associates with clinical outcomes of breast cancer. Frontiers in Oncology*,* 11*,* 628677; 34395236 10.3389/fonc.2021.628677 PMC8356635

[7] Guo, Z., Zhang, H., Fu, Y., Kuang, J., Zhao, B. et al. (2023). Cancer-associated fibroblasts induce growth and radioresistance of breast cancer cells through paracrine IL-6. Cell Death Discovery*,* 9 *(*1*),* 6; 36635302 10.1038/s41420-023-01306-3 PMC9837084

[8] Kumari, N., Dwarakanath, B. S., Das, A., Bhatt, A. N. (2016). Role of interleukin-6 in cancer progression and therapeutic resistance. Tumor Biology*,* 37 *(*9*),* 11553–11572; 27260630 10.1007/s13277-016-5098-7

[9] Martinez-Outschoorn, U. E., Sotgia, F., Lisanti, M. P. (2015). Caveolae and signalling in cancer. Nature Reviews Cancer*,* 15 *(*4*),* 225–237. 25801618 10.1038/nrc3915

[10] Martinez-Outschoorn, U. E., Pavlides, S., Whitaker-Menezes, D., Daumer, K. M., Milliman, J. N. et al. (2010). Tumor cells induce the cancer associated fibroblast phenotype via caveolin-1 degradation: Implications for breast cancer and DCIS therapy with autophagy inhibitors. Cell Cycle*,* 9 *(*12*),* 2423–2433. 20562526 10.4161/cc.9.12.12048

[11] Jena, B. C., Das, C. K., Banerjee, I., Bharadwaj, D., Majumder, R. et al. (2022). TGF-β1 induced autophagy in cancer associated fibroblasts during hypoxia contributes EMT and glycolysis via MCT4 upregulation. Experimental Cell Research*,* 417 *(*1*),* 113195; 35561786 10.1016/j.yexcr.2022.113195

[12] Guido, C., Whitaker-Menezes, D., Capparelli, C., Balliet, R., Lin, Z. et al. (2012). Metabolic reprogramming of cancer-associated fibroblasts by TGF-β drives tumor growth: Connecting TGF-β signaling with “Warburg-like” cancer metabolism and L-lactate production. Cell Cycle*,* 11 *(*16*),* 3019–3035. 22874531 10.4161/cc.21384 PMC3442913

[13] Brooks, G. A. (2018). The science and translation of lactate shuttle theory. Cell Metabolism*,* 27 *(*4*),* 757–785; 29617642 10.1016/j.cmet.2018.03.008

[14] Dias, A. S., Almeida, C. R., Helguero, L. A., Duarte, I. F. (2019). Metabolic crosstalk in the breast cancer microenvironment. European Journal of Cancer*,* 121*,* 154–171. 31581056 10.1016/j.ejca.2019.09.002

[15] Ayala, G., Morello, M., Frolov, A., You, S., Li, R. et al. (2013). Loss of caveolin-1 in prostate cancer stroma correlates with reduced relapse-free survival and is functionally relevant to tumour progression. The Journal of Pathology*,* 231 *(*1*),* 77–87. 23729330 10.1002/path.4217 PMC3978784

[16] Zhao, X., He, Y., Gao, J., Fan, L., Li, Z. et al. (2013). Caveolin-1 expression level in cancer associated fibroblasts predicts outcome in gastric cancer. PLoS One*,* 8 *(*3*),* e59102; 23527097 10.1371/journal.pone.0059102 PMC3602462

[17] Elwakeel, E., Weigert, A. (2021). Breast cancer CAFs: Spectrum of phenotypes and promising targeting avenues. International Journal of Molecular Sciences*,* 22 *(*21*),* 1–20. 10.3390/ijms222111636 PMC8583860 34769066

[18] Linares, J., Marín-Jiménez, J. A., Badia-Ramentol, J., Calon, A. (2021). Determinants and functions of CAFs secretome during cancer progression and therapy. Frontiers in Cell and Developmental Biology*,* 8*,* 1–19. 10.3389/fcell.2020.621070 PMC7862334 33553157

[19] Mostafavi, S., Zalpoor, H., Hassan, Z. M. (2022). The promising therapeutic effects of metformin on metabolic reprogramming of cancer-associated fibroblasts in solid tumors. Cellular & Molecular Biology Letters*,* 27 *(*1*),* 58. 35869449 10.1186/s11658-022-00356-2 PMC9308248

[20] Zhang, J., Li, G., Chen, Y., Fang, L., Guan, C. et al. (2017). Metformin inhibits tumorigenesis and tumor growth of breast cancer cells by upregulating miR-200c but downregulating AKT2 expression. Journal of Cancer*,* 8 *(*10*),* 1849–1864; 28819383 10.7150/jca.19858 PMC5556649

[21] Kheirandish, M., Mahboobi, H., Yazdanparast, M., Kamal, W., Kamal, M. A. (2018). Anti-cancer effects of metformin: Recent evidences for its role in prevention and treatment of cancer. Current Drug Metabolism*,* 19 *(*9*),* 793–797. 29663879 10.2174/1389200219666180416161846

[22] Charan, J., Kantharia, N. D. (2013). How to calculate sample size in animal studies? Journal of Pharmacology & Pharmacotherapeutics*,* 4 *(*4*),* 303–306. 24250214 10.4103/0976-500X.119726 PMC3826013

[23] Rizvi, F., Shaukat, L., Azhar, A., Jafri, A., Aslam, U. et al. (2021). Preclinical meritorious anticancer effects of metformin against breast cancer: An *in vivo* trial. Journal of Taibah University Medical Science*,* 16 *(*4*),* 504–512; 34408607 10.1016/j.jtumed.2021.02.006 PMC8348326

[24] Thies, S., Langer, R. (2013). Tumor regression grading of gastrointestinal carcinomas after neoadjuvant treatment. Frontiers in Oncology*,* 3*,* 262; 24109590 10.3389/fonc.2013.00262 PMC3791673

[25] Abbasi, A., Pakravan, N., Hassan, Z. M. (2021). Hyaluronic acid optimises therapeutic effects of hydrogen peroxide-induced oxidative stress on breast cancer. Journal of Cellular Physiology*,* 236 *(*2*),* 1494–1514. 32740942 10.1002/jcp.29957

[26] Pavlova, N. N., Thompson, C. B. (2016). The emerging hallmarks of cancer metabolism. Cell Metabolism*,* 23 *(*1*),* 27–47; 26771115 10.1016/j.cmet.2015.12.006 PMC4715268

[27] Martins, D., Beça, F. F., Sousa, B., Baltazar, F., Paredes, J. et al. (2013). Loss of caveolin-1 and gain of MCT4 expression in the tumor stroma: Key events in the progression from an in situ to an invasive breast carcinoma. Cell Cycle*,* 12 *(*16*),* 2684–2690. 23907124 10.4161/cc.25794 PMC3865058

[28] Shao, S., Qin, T., Qian, W., Yue, Y., Xiao, Y. et al. (2020). Positive feedback in Cav-1-ROS signalling in PSCs mediates metabolic coupling between PSCs and tumour cells. Journal of Cellular and Molecular Medicine*,* 24 *(*16*),* 9397–9408; 32633891 10.1111/jcmm.15596 PMC7417714

[29] Xue, L., Liu, J., Xie, J., Luo, J. (2021). Prognostic value of SLC16A3(MCT4) in lung adenocarcinoma and its clinical significance. International Journal of General Medicine*,* 14*,* 8413–8425. 34819749 10.2147/IJGM.S337615 PMC8607606

[30] Zhao, Y., Zhao, B., Yan, W. H., Xia, Y., Wang, Z. H. et al. (2021). Integrative analysis identified MCT4 as an independent prognostic factor for bladder cancer. Frontiers in Oncology*,* 11*,* 1–10. 10.3389/fonc.2021.704857 PMC8426349 34513685

[31] Yuan, C., Zhang, J., Lou, J., Wang, S., Jiang, Y. et al. (2021). Comprehensive analysis of monocarboxylate transporter 4 (MCT4) expression in breast cancer prognosis and immune infiltration via integrated bioinformatics analysis. Bioengineered*,* 12 *(*1*),* 3850–3863; 34269158 10.1080/21655979.2021.1951928 PMC8806482

[32] Ren, L., Zhou, P., Wu, H., Liang, Y., Xu, R. et al. (2021). Caveolin-1 is a prognostic marker and suppresses the proliferation of breast cancer. Translational Cancer Research*,* 10 *(*8*),* 3797–3810. 35116679 10.21037/tcr-21-1139 PMC8798413

[33] Kim, H. J., Kwon, H., Lee, J. W., Kim, H. J., Lee, S B. et al. (2015). Metformin increases survival in hormone receptor-positive, HER2-positive breast cancer patients with diabetes. Breast Cancer Research*,* 17 *(*1*),* 1–14. 25935404 10.1186/s13058-015-0574-3 PMC4504447

[34] Chung, Y. C., Chang, C. M., Wei, W. C., Chang, T. W., Chang, K. J. et al. (2018). Metformin-induced caveolin-1 expression promotes T-DM1 drug efficacy in breast cancer cells. Scientific Reports*,* 8 *(*1*),* 3930; 29500444 10.1038/s41598-018-22250-8 PMC5834501

[35] Takasaka, N., Araya, J., Kurita, Y., Kobayashi, K., Ito, S. et al. (2014). Metformin inhibits TGF-b-induced myofibroblast differentiation through AMPK activation. European Respiratory Journal*,* 44 *(*Suppl 58*),* P3854.

[36] Shi, X., Yang, J., Deng, S., Xu, H., Wu, D. et al. (2022). TGF-β signaling in the tumor metabolic microenvironment and targeted therapies. Journal of Hematology & Oncology*,* 15 *(*1*),* 135. 36115986 10.1186/s13045-022-01349-6 PMC9482317

[37] Goulet, C. R., Champagne, A., Bernard, G., Vandal, D., Chabaud, S. et al. (2019). Cancer-associated fibroblasts induce epithelial-mesenchymal transition of bladder cancer cells through paracrine IL-6 signalling. BMC Cancer*,* 19 *(*1*),* 137. 30744595 10.1186/s12885-019-5353-6 PMC6371428

[38] Porporato, P. E., Filigheddu, N., Pedro, J. M. B. S., Kroemer, G., Galluzzi, L. (2018). Mitochondrial metabolism and cancer. Cell Research*,* 28 *(*3*),* 265–280. 29219147 10.1038/cr.2017.155 PMC5835768

[39] Andrzejewski, S., Siegel, P. M., St-Pierre, J. (2018). Metabolic profiles associated with metformin efficacy in cancer. Frontiers in Endocrinology*,* 9*,* 372; 30186229 10.3389/fendo.2018.00372 PMC6110930

